# A persistent northern boundary of Indian Summer Monsoon precipitation over Central Asia during the Holocene

**DOI:** 10.1038/srep25791

**Published:** 2016-05-13

**Authors:** Arne Ramisch, Gregori Lockot, Torsten Haberzettl, Kai Hartmann, Gerhard Kuhn, Frank Lehmkuhl, Stefan Schimpf, Philipp Schulte, Georg Stauch, Rong Wang, Bernd Wünnemann, Dada Yan, Yongzhan Zhang, Bernhard Diekmann

**Affiliations:** 1 Alfred Wegener Institute Helmholtz Centre for Polar and Marine Research, Potsdam, Germany; 2 Institute of Geographical Science, Free University of Berlin, Berlin, Germany; 3 Institute of Geography, Friedrich-Schiller-University Jena, Jena, Germany; 4 Alfred Wegener Institute Helmholtz Centre for Polar and Marine Research, Bremerhaven, Germany; 5 Department of Geography, RWTH Aachen University, Aachen, Germany; 6 Nanjing Integrated Centre for Earth System Science, Nanjing, China; 7 School of Geography and Oceanography, Nanjing University, Nanjing, China; 8 Institute of Earth and Environmental Science, University of Potsdam, Potsdam-Golm, Germany

## Abstract

Extra-tropical circulation systems impede poleward moisture advection by the Indian Summer Monsoon. In this context, the Himalayan range is believed to insulate the south Asian circulation from extra-tropical influences and to delineate the northern extent of the Indian Summer Monsoon in central Asia. Paleoclimatic evidence, however, suggests increased moisture availability in the Early Holocene north of the Himalayan range which is attributed to an intensification of the Indian Summer Monsoon. Nevertheless, mechanisms leading to a surpassing of the Himalayan range and the northern maximum extent of summer monsoonal influence remain unknown. Here we show that the Kunlun barrier on the northern Tibetan Plateau [~36°N] delimits Indian Summer Monsoon precipitation during the Holocene. The presence of the barrier relocates the insulation effect 1,000 km further north, allowing a continental low intensity branch of the Indian Summer Monsoon which is persistent throughout the Holocene. Precipitation intensities at its northern extent seem to be driven by differentiated solar heating of the Northern Hemisphere indicating dependency on energy-gradients rather than absolute radiation intensities. The identified spatial constraints of monsoonal precipitation will facilitate the prediction of future monsoonal precipitation patterns in Central Asia under varying climatic conditions.

Substantial progress was made in understanding influences on the poleward extent of the Indian Summer Monsoon (ISM). It is now widely believed that the Asian subtropical landmass causes positive energy fluxes in boreal summer into the atmosphere^1^. The resulting convergence of air masses over continental Asia may be regarded as a north shift of the inner tropical convergence zone (ITCZ) which causes advection of warm and moist air masses from the Indian Ocean poleward from a south-westerly direction^2^. The poleward motion of the ITCZ is impeded by the advection of cold and dry air masses from mid-latitude oceans through the westerly circulation^3^,^4^ which tends to supress convection due to its low subcloud moist static energy. This so-called *ventilation effect*^4^ is blocked by the orography of the Indian subcontinent: the narrow barrier of the Himalayans insulates the high moist static energy of ISM circulation from the low moist static energy of the westerly circulation, independent of the high-elevated Tibetan Plateau north of the barrier^5^. Hence, the Himalayan range delineates the northern boundary of ISM precipitation over continental central Asia (see Fig. 1).

However, paleoclimatic evidence from the Tibetan Plateau challenges the hypothesis of a temporally stable monsoon boundary. Many paleoenvironmental archives indicate a dramatic rise in moisture availability after Termination 1 approximately 12 ka ago^6^,^7^,^8^. After the early Holocene moisture optimum, moisture availability and precipitation intensities decreased in a gradual fashion following boreal summer insolation^6^, though lagging insolation intensities in different magnitude^6^,^9^. It is generally believed that decreasing insolation led to a decreased positive energy influx into the atmosphere over the central Asian continent, thus diminishing the thermal land-sea gradient and relocating the ITCZ further south^10^. This relocation is also observed to the west of the Tibetan Plateau in speleothem records from the Arabian Peninsula^11^ and to the east in the East Asian Summer Monsoon branch as recorded by lacustrine sediments from eastern China^12^,^13^. Hence, a variable boundary of monsoonal precipitation is the only mechanism capable to explain the observed paleo-moisture evolution over the Tibetan Plateau. However, the maximum northward ISM extent remains unknown.

To reconstruct monsoonal precipitation variations north of the Himalayan barrier we analysed sediment dynamics on two mountain ranges at the northern fringe of the Tibetan Plateau: The Kunlun and Burhan Buda mountain range (Fig. 2a), both normal to ISM trajectories. The mountain ranges delineate the boundary between the Tibetan Plateau and the internally-drained Qaidam Basin and adjacent central Asian deserts in the North. At this latitude, archives of paleoenvironmental changes become sparse because of the prevailing aridity and high altitude. One of the few archives is Lake Heihai, situated in an intermontane basin in between the two mountain ranges. Lake Heihai (Fig. 2) is a mesohaline, open lake system with an area of 38 km^2^ and maximum depth of 22.5 m. It constitutes a sink of two sediment cascades originating in the Kunlun range in the south and the Burhan Buda range in the north. We sampled 58 active and inactive streams on alluvial fans (corresponding to ~990 km^2^ drainage area) to study fluvial sediment sources within the basin.

## Results and Discussion

**Figure 2:**
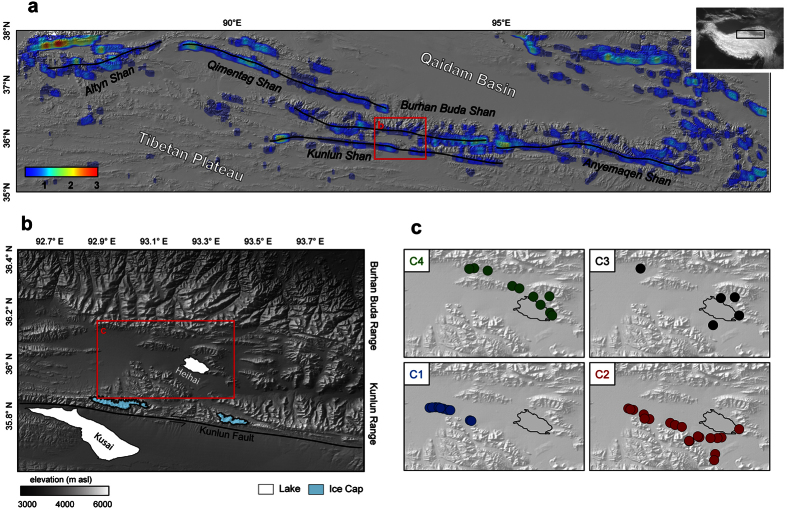
Sediment sources in the central Kunlun fault system. (**a**) Regional overview and the North Tibetan precipitation barrier. Areas with similar or higher orographic forcing on southerly air masses (as compared to the study area) are indicated by colours as factor of higher forcing (supplementary information). (**b**) Topographic map of the study area. (**c**) Results of cluster analysis for reference samples on alluvial fans in the study area. Circles indicate sample location. Colours indicate dominant membership (μ > 0.5) of samples to mineralogical cluster C1 to C4 (supplementary information). Maps were created using ArcGIS 10.1 (www.esri.com) and Adobe Illustrator CS 4 (www.adobe.com).

The application of a fuzzy c-means cluster analysis on the minerogenic content of the reference samples reveals 4 dominant sources of fluvial sediment supply (Fig. 2b, see supplementary information). Cluster C1 is situated on alluvial fans originating from a 50 km^2^ large ice cap on the Kunlun range, suggesting a seasonal activation through glacio-fluvial meltwaters. Cluster 2, 3 and 4 represent fluvial sediments originating from foothills of the Kunlun range (Cluster C2) and Burhan Buda range (C3 and C4). Due to the lack of morphological evidences for former glaciation we assume predominantly precipitation generated runoff for these sub-catchments. Cluster C3 and C4 mainly differ in their mean distance to the lake basin which is ~4 km (C3) and ~11.5 km (C4), respectively.

**Figure 3:**
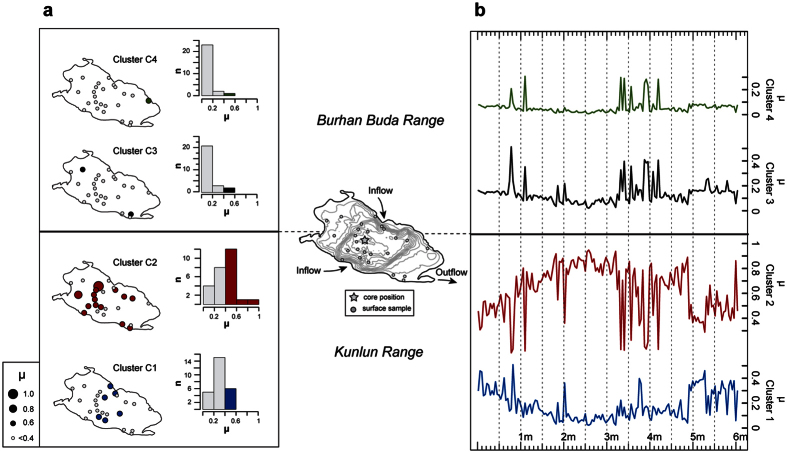
Mineralogical similarity of lacustrine sediments to sediment sources. Surface and core sample locations are shown in the center of the figure. (**a**) Membership degrees of lake surface samples to mineralogical cluster C1 to C4. Lake sketch indicates the position (circle) and membership degree (circle size) of individual samples to cluster center C1 to C4. Histograms indicate the total number (n) of lake surface samples in membership classes (μ with 0.2 bin) to a respective cluster center. (**b**) Membership degrees of core samples to mineralogical cluster C1 to C4. Sample memberships are plotted against depth in a sediment core. Maps were created using ArcGIS 10.1 (www.esri.com) and Adobe Illustrator CS 4 (www.adobe.com).

To estimate the contribution of each mentioned sediment source in the basin to the present-day sediment supply of Lake Heihai we sampled the recent lake floor at 28 positions. The similarity of the lacustrine detrital fraction to sediment provinces has been obtained by calculating fuzzy membership degrees [μ] of a samples mineralogical composition to Cluster C1 to C4 (Fig. 3a). Values of μ are in a theoretical range of 1 (for complete membership) and 0 (for absent membership). Results suggest that the detrital fraction of lake sediments mainly originate from the Kunlun range with almost equal importance of glacio-fluvial (mean μ = 0.31, C1) and precipitation induced (mean μ = 0.39, C2) runoff (Fig. 3). The precipitation induced sediment supply from the Burhan Buda range is low and its relative importance decreases with increasing northward distance to the Kunlun range from μ = 0.2 (C3) to μ = 0.1 (C4).

**Figure 4:**
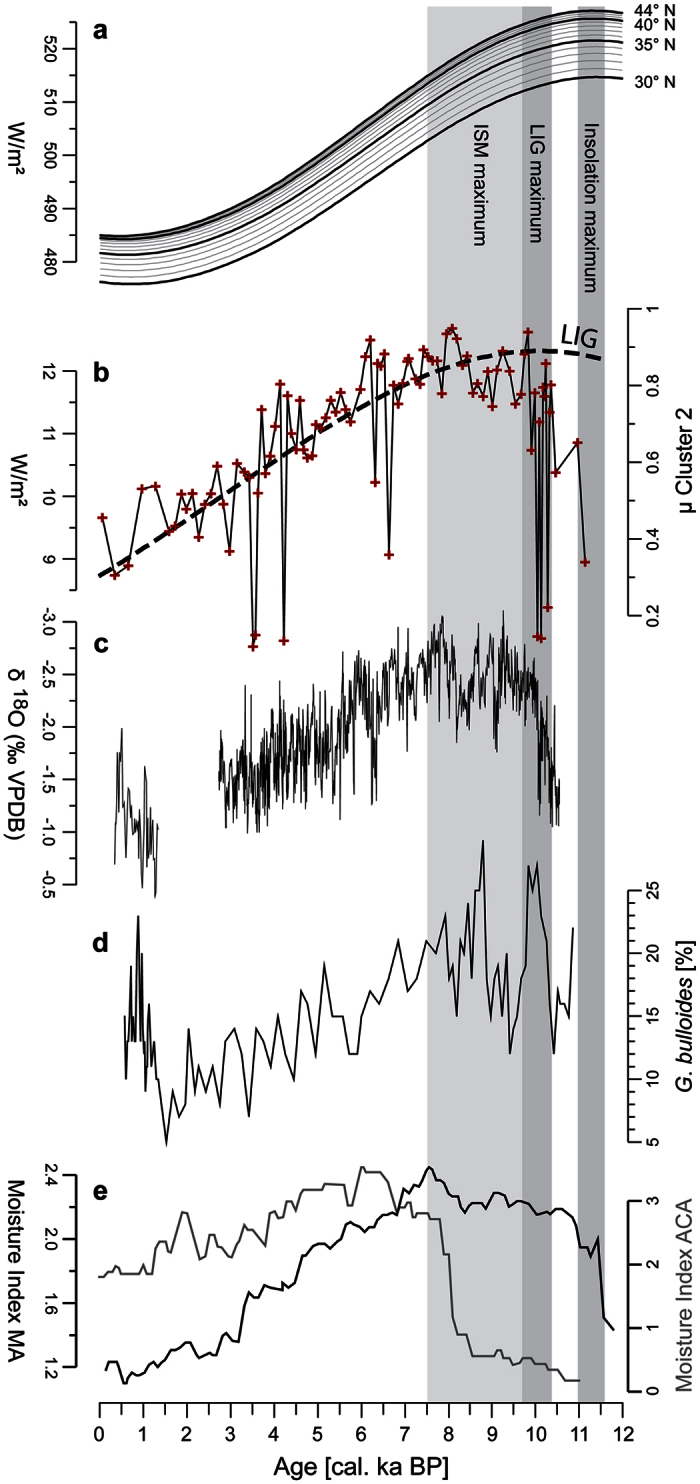
Radiative forcing and ISM intensity variations. (**a**) Boreal summer insolation intensities (summer solstice) between 30° and 44°N^31^. (**b**) Proxy for ISM precipitation variation in its northernmost location as determined in this study. Black dashed line indicates the Holocene evolution of the Latitudinal Insolation Gradient (LIG) between 30°N and 44°N. (**c**) Proxy for ISM precipitation intensity variation as recorded by speleothems in Oman^14^. (**d**) Proxy for ISM wind strength over the Arabian Sea^15^. (**e**) Moisture evolution in paleoenvironmental archives throughout monsoonal Asia (MA)^7^ and its out-of-phase relationship to the moisture evolution in arid central Asia (ACA)^23^.

For a temporal analysis, a sediment core was recovered from the deepest part of the lake basin. Our lake record extends back into the Late Pleistocene (supplementary information). The asymmetric sediment supply between the southern and northern range is persistent throughout the temporal coverage of the sediment record (Fig. 3b). Mean mineralogical similarities of core samples to sediment sources activated by precipitation drop from μ = 0.67 for the southern range to 0.12–0.05 on the northern range. Indicating linkages to boreal summer insolation, southern sediment sources show peak activation in the Early Holocene (10 to ~7.5 cal. ka BP) and a gradual decrease in the mid to Late Holocene (Fig. 4a,b). The trend tends to be synchronous to decreasing Holocene ISM intensities recorded by Oman speleothems^11^,^14^ (Fig. 4c), upwelling in the Arabian Sea induced by monsoonal winds^15^ (Fig. 4d) and continental archives in monsoonal Asia^6^,^7^ (Fig. 4e). The decrease is asynchronous to the moisture evolution of the East Asian Summer Monsoon^6^,^12^ and balanced by a compositional change to glacio-fluvial sediment sources at Lake Heihai (Fig. 3b). We thus assume a dominant link of summer rainfall on the Kunlun range to ISM precipitation.

We attribute the low intensity of fluvial sediment supply from the Burhan Buda range to a barrier effect of the Kunlun range. The orography of the Kunlun range forces moisture bearing air masses migrating on ISM trajectories to a sudden ascend, leading to intensified orographic precipitation. Although the Burhan Buda range imposes a similar orographic forcing on southerly air masses (supplementary information), the remaining atmospheric moisture is insufficient for orographic precipitation to occur. The orography of the Kunlun range thus delineates the northern boundary of ISM precipitation in the central Kunlun fault system. A comparison with the orographic structure of the northern Tibetan Plateau (supplementary information) revealed an extent of the barrier with similar or higher orographic forcing on air masses for ~1,200 km from 86° to 105°E (Fig. 2a). Obviously, the barrier is blocking nearly 90% of summer precipitation as calculated from observational and reanalysed climate data (supplementary information). There are no additional atmospheric moisture sources in the central Asian desert belt north of the barrier which could enable a reactivation of summer monsoonal precipitation. Therefore, we conclude that the barrier forms a continuous boundary of ISM precipitation on the Tibetan Plateau (Fig. 2a). Although these mountain ranges have never been associated with the spatial extent of ISM precipitation, numerous studies focussing on the northern extent of ISM precipitation based on observational data^16^,^17^ paleo-climatic reconstructions^6^ and paleo-climate modelling^18^ are in good agreement with the latitudinal extent of the barrier at ~36°N. The effect even might extent further back in time, since the Kunlun Mountains cause aridity in the Qaidam Basin since 22 Ma^19^. Furthermore, an intensified uplift associated with the Kunlun Range in the Mid-Pleistocene caused an intensification of arid conditions in the Asian inland^20^. While ISM precipitation dominates in the central parts, uncertainty remains about the influence of the East Asian Monsoon on precipitation patterns in the east of the barrier.

Based on our results we suggest that ISM precipitation continuously prevails between the Himalayan and Kunlun range in a low intensity branch. The branch is persistent throughout the Holocene. The persistency implies that the actual insulation between air masses of high and low moist static energy, formerly attributed to the Himalayans^2^,^5^, actually occurs at ~36°N. This assumption is reinforced by the asynchronous Holocene moisture evolution south and north of the North Tibetan barrier as evidenced by paleoenvironmental archives throughout monsoonal Asia^6^,^7^ and arid central Asia^21^,^22^,^23^ south and north of the barrier respectively (see Fig. 4e). The out-of-phase relationship is ascribed to a predominant westerly influence on the moisture evolution of arid central Asia^23^, reaffirming the delineation of major circulation systems at the northern Tibetan barrier. Thus, our lake record mirrors the northernmost intensity variations of dominant ISM influence.

If the thermal land-sea contrast is responsible for the ocean-to-continent advection of high moist static energy, which forcing mechanism continues to set the inner-continental poleward advection of ISM air masses in motion? Monsoonal rainfalls can occur in high latitudes under idealized conditions as suggested by climate models which exclude impeding mechanisms such as the ventilation effect and Rossby-Wave dynamics^4^. This suggests that in the absence of impeding mechanisms, radiative forcing drives the spatial extent of monsoonal rainfalls. However, our record lags the absolute intensity of boreal summer insolation with a time lag of ~2 ka, a phenomenon observed in the majority of Holocene monsoonal records^9^,^24^. This phase lag of monsoonal strength to orbital forcing is partly attributed to a delayed maximum latent heat export from the Southern Indian Ocean resulting from differentiated northern and southern hemispheric insolation forcing^24^,^25^,^26^. However, while explaining monsoonal intensity variations in Southern Asia to a latitude of ~30°^25^, this mechanism is unlikely to account for an inner-continental northward advection of monsoonal rainfalls north of the Himalayan range during the Holocene. Interestingly, our record closely follows the Holocene latitudinal insolation gradient between daily summer insolation rates of the Himalayan barrier (30°) and the latitude of mean daily peak insolation (44°N) (Fig. 4b). Absolute intensities converge proportionally between the latitudes from the Early to Late Holocene (Fig. 4a) synchronous to decreasing precipitation intensities at the northern ISM boundary. We assume that the impact of differentiated radiative forcing on the inner-continental thermal regime^27^ drives the inner-continental northward migration of the Tibetan ISM branch on centennial to millennial time scales. The decrease in thermal poleward forcing led to decreased moist static energy advection, limiting, but not excluding convergence in higher latitudes as evidenced by continuous but decreasing Holocene precipitation intensities at the northern ISM barrier.

Our results have great implications for future climate scenarios and modelling approaches in central and high Asia. A projected increase in global surface temperatures in response to anthropogenic forcing is accompanied by increasing monsoonal precipitations^28^ impacting especially South Asia^2^. However, climate models often underestimate the Tibetan ISM branch because of its low intensity. Our record shows that changes in monsoonal strength have an immediate impact on precipitation in the Tibetan ISM branch in its entire northward extent. An increase in precipitation on the Tibetan Plateau would affect socio-economically important environmental subsystems such as glaciers, rivers and vegetation^29^. Any change in ISM precipitation dynamics, however, would be limited to ~36°N and unlikely affect climate in arid central Asia. The limited spatial extent of ISM precipitation in central Asia may thus be used to validate climate models. Furthermore, we suggest that besides inter-hemispheric gradients^30^ and absolute forcing mechanisms like global temperatures or insolation, their inner-hemispheric distribution must be taken into account for predicting future inner-continental ISM changes.

## Methods

Basin reference samples were collected at 58 locations. The sampling strategy followed the intention to infer a comprehensive mineralogical fingerprint of sediments within the drainage basin. To minimize grain size differences in between reference samples and core sediments which could lead to a systematic grain size bias (see supplementary information), we only sampled the fine fraction (sand to clay) of fluvial sediments in former river bars and pools of inactive and active streams. The lake surface was sampled with a UWITEC gravity corer and Bottom Grab Sampler. Gravity cores were sampled only on their surfaces (0–1 cm). We retrieved three piston cores which overlap to a total length of 6.03 m from the deepest part (water depth of 22.4 m) of the lake basin. The cores were continuously sampled in a 4 cm interval. Basin, lake and core samples were freeze-dried and subsequently grinded 5 minutes at 3600 rpm with a vibration disc mill to a grain size approximately <4 μm. Subsequently, the mineralogy was estimated on the powdered material using a PANalytical Empyrean diffractometer. The 2θ range (2–52°) was analyzed in 0.01° steps with a copper Kα X-Ray tube. XRD measurements were carried out at the Alfred-Wegener-Institute in Bremerhaven. We applied a fuzzy c-means cluster analysis to determine major sediment sources within the drainage basin and compared their similarity to lacustrine sediments via fuzzy membership degrees (supplementary Information). Organic material in form of plant macro remains (*Potamogeton* spec.) was sampled according to their occurrence within the core. To estimate the modern reservoir effect of the lake water we additionally dated three recently living species of *Potamogeton* spec. during the field campaign in September 2012. All ^14^C samples were freeze dried and send to Poznan Radiocarbon Laboratory (Poznan, Poland) and Beta Analytics Inc. (Miami, Florida, USA) for AMS-^14^C dating. The methodology of establishing an age-depth relationship for the piston core was recently published elsewhere^32^ and is described in greater detail in the supplementary information. Supplementary data is also available at https://doi.org/10.1594/PANGAEA.860017.

## Additional Information

**How to cite this article**: Ramisch, A. *et al*. A persistent northern boundary of Indian Summer Monsoon precipitation over Central Asia during the Holocene. *Sci. Rep.* **6**, 25791; doi: 10.1038/srep25791 (2016).

## Supplementary Material

Supplementary Information

## Figures and Tables

**Figure 1:**
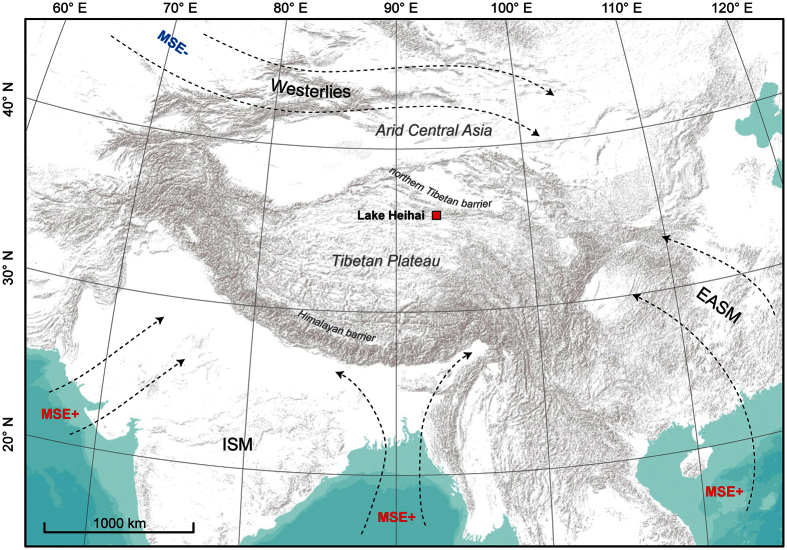
Conceptual model of circulation patterns over central Asia during boreal summer. Dashed arrows depict idealized boreal summer circulation over central Asia. MSE+ and MSE− refer to the advection of high and low moist static energy respectively. For detailed studies of present day moisture transport over the Tibetan Plateau during the monsoon season the reader is referred to^16^ and^33^. Maps were created using ArcGIS 10.1 (www.esri.com) with the World Terrain Basemap (Esri, USGS, NOAA) and Adobe Illustrator CS 4 (www.adobe.com).

## References

[b1] Chou C. & Neelin J. D. Mechanisms Limiting the Northward Extent of the Northern Summer Monsoons over North America, Asia, and Africa. J. Clim. 16, 406–425 (2003).

[b2] Turner A. G. & Annamalai H. Climate change and the South Asian summer monsoon. Nature Clim. Change 2, 587–595 (2012).

[b3] Privé N. C. & Plumb R. A. Monsoon dynamics with interactive forcing. Part II: Impact of eddies and asymmetric geometries. J. Atmos. Sci. 64, 1431–1442 (2007).

[b4] Chou C., Neelin J. D. & Su H. Ocean-atmosphere-land feedbacks in an idealized monsoon. Q. J. R. Meteorol. Soc. 127, 1869–1891 (2001).

[b5] Boos W. R. & Kuang Z. Dominant control of the South Asian monsoon by orographic insulation versus plateau heating. Nature 463, 218–222 (2010). 20075917 10.1038/nature08707

[b6] Wang Y., Liu X. & Herzschuh U. Asynchronous evolution of the Indian and East Asian Summer monsoon indicated by Holocene moisture patterns in monsoonal central Asia. Earth-Science Reviews 103, 135–153 (2010).

[b7] Herzschuh U. Palaeo-moisture evolution in monsoonal Central Asia during the last 50,000 years. Quat. Sci. Rev. 25, 163–178 (2006).

[b8] Doberschütz . Monsoonal forcing of Holocene paleoenvironmental change on the central Tibetan Plateau inferred using a sediment record from Lake Nam Co (Xizang, China). J Paleolimnol 51, 253–266 (2014).

[b9] Overpeck J., Anderson D., Trumbore S. & Prell W. The southwest Indian Monsoon over the last 18 000 years. Clim. Dyn. 12, 213–225 (1996).

[b10] Wanner H. . Mid- to Late Holocene climate change: an overview. Quat. Sci. Rev. 27, 1791–1828 (2008).

[b11] Fleitmann D. . Holocene ITCZ and Indian monsoon dynamics recorded in stalagmites from Oman and Yemen (Socotra). Quat. Sci. Rev. 26, 170–188 (2007).

[b12] An Z. . Asynchronous Holocene optimum of the East Asian monsoon. Quat Sci. Rev. 19, 743–762 (2000).

[b13] Yancheva G. . Influence of the intertropical convergence zone on the East Asian monsoon. Nature 445, 74–77 (2007). 17203059 10.1038/nature05431

[b14] Fleitmann D. . Holocene Forcing of the Indian Monsoon Recorded in a Stalagmite from Southern Oman. Science 300, 1737–1739 (2003). 12805545 10.1126/science.1083130

[b15] Gupta A. K., Anderson D. M. & Overpeck J. T. Abrupt changes in the Asian southwest monsoon during the Holocene and their links to the North Atlantic Ocean. Nature 421, 354–357 (2003). 12540924 10.1038/nature01340

[b16] Conroy J. L. & Overpeck J. T. Regionalization of Present-Day Precipitation in the Greater Monsoon Region of Asia. J. Clim. 24, 4073–4095 (2011).

[b17] Tian L., Masson-Delmotte V., Stievenard M., Yao T. & Jouzel J. Tibetan Plateau summer monsoon northward extent revealed by measurements of water stable isotopes. J. Geophys. Res. 106, 28081–28088 (2001).

[b18] Jiang D., Tian Z. & Lang X. Mid-Holocene net precipitation changes over China: model-data comparison. Quat. Sci. Rev. 82, 104–120 (2013).

[b19] Sobel E. R., Hilley G. E. & Strecker M. R. Formation of internally drained contractional basins by aridity-limited bedrock incision. J. Geophys. Res. 108, 1–23 (2003).

[b20] Han W., Fang X., Ye C., Teng X. & Zhang T. Tibet forcing Quaternary stepwise enhancement of westerly jet and central Asian aridification: carbonate isotope records from deep drilling in the Qaidam salt playa, NE Tibet. Global and Planetary Change, 68–75 (2014).

[b21] Lu H. . Holocene climatic changes revealed by aeolian deposits from the Qinghai Lake area (northeastern Qinghai-Tibetan Plateau) and possible forcing mechanisms. The Holocene 21, 297–304 (2010).

[b22] He Y. . Late Holocene coupled moisture and temperature changes on the northern Tibetan Plateau. Quat. Sci. Rev. 80, 47–57 (2013).

[b23] Chen F. . Holocene moisture evolution in arid central Asia and its out-of-phase relationship with Asian monsoon history. Quat. Sci. Rev. 27, 351–364 (2008).

[b24] Clemens S. C., Prell W. L. & Sun Y. Orbital‐scale timing and mechanisms driving Late Pleistocene Indo‐Asian summer monsoons: Reinterpreting cave speleothem δ18O. Paleoceanography 25, PA4207 (2010).

[b25] Liu X., Liu Z., Kutzbach J. E., Clemens S. C. & Prell W. L. Hemispheric Insolation Forcing of the Indian Ocean and Asian Monsoon: Local versus Remote Impacts. J. Clim. 19, 6195–6208 (2006).

[b26] Caley T. . New Arabian Sea records help decipher orbital timing of Indo-Asian monsoon. Earth Planet. Sci. Lett. 308.3, 433–444 (2011).

[b27] Davis B. A. S. & Brewer S. Orbital forcing and role of the latitudinal insolation/temperature gradient. Clim. Dyn. 32, 143–165 (2009).

[b28] Hu Z. Z., Latif M., Roeckner E. & Bengtsson L. Intensified Asian summer monsoon and its variability in a coupled model forced by increasing greenhouse gas concentrations. Geophys. Res. Lett. 27, 2681–2684 (2000).

[b29] Immerzeel W. W., van Beek L. P. H. & Bierkens M. F. P. Climate Change Will Affect the Asian Water Towers, Science 328, 1382–1385 (2010). 20538947 10.1126/science.1183188

[b30] Zisheng A. . Glacial-Interglacial Indian Summer Monsoon Dynamics. Science 333, 719–723 (2011). 21817044 10.1126/science.1203752

[b31] Laskar J. . A long term numerical solution for the insolation quantities of the Earth. Astron. Astrophys. 428, 261–285 (2004).

[b32] Lockot G. . A process- and provenance based attempt to unravel inconsistent radiocarbon chronologies in lake sediments: an example from Lake Heihai, North Tibetan Plateau. Radiocarbon 57, 1–17 (2015).

[b33] Maussion F. . Precipitation Seasonality and Variability over the Tibetan Plateau as Resolved by the High Asia Reanalysis. J. Clim. 27, 1910–1927 (2014).

