# Supplementary material for: A persistent northern boundary of Indian Summer Monsoon precipitation over Central Asia during the Holocene
Source: Sci Rep. 2016 May 13;6:25791. doi: 10.1038/srep25791 (PMC4865755; doi:10.1038/srep25791)
Supplement: Supplementary Information [file srep25791-s1.pdf]

# *Supplementary Information*

A persistent northern boundary of Indian Summer Monsoon precipitation over Central Asia during the Holocene

**Authors**: Arne Ramisch[1], Gregori Lockot[2], Torsten Haberzettl[3], Kai Hartmann[2], Gerhard Kuhn[4], Frank Lehmkuhl[5], Stefan Schimpf[1], Philipp Schulte[5], Georg Stauch[5], Rong Wang[1], Bernd Wünnemann[2,6], Dada Yan[6], Yongzhan Zhang[7], Bernhard Diekmann[1,8]

*[1] Alfred Wegener Institute Helmholtz Centre for Polar and Marine Research, Potsdam, Germany*

*[2] Institute of Geographical Science, Free University of Berlin, Berlin, Germany*

*[3] Institute of Geography, Friedrich-Schiller-University Jena, Jena, Germany*

*[4] Alfred Wegener Institute Helmholtz Centre for Polar and Marine Research, Bremerhaven, Germany*

*[5] Department of Geography, RWTH Aachen University, Aachen, Germany*

*[6] Nanjing Integrated Centre for Earth System Science, Nanjing, China*

*[7] School of Geography and Oceanography, Nanjing University, Nanjing, China*

*[8] Institute for Earth and Environmental Science, University of Potsdam, Potsdam-Golm, Germany*

# 1 Supplementary Methods

## 1.1 Mineralogical Analysis

X-ray diffractometry was used to obtain data on the composition and abundances of minerogenic components in the source areas and the lake sediments [34]. Freeze-dried and milled bulk sediment samples were analysed by XRD, using a (PHILIPS, Netherlands) PW1820 goniometer (40 kV, 40 mA, from 3 to 100°, step-rate 0.05° Co k-alpha radiation). Mineral concentrations were calculated semi-quantitatively from main peak area intensities of mineral species in the diffractograms (Supplementary Table 1). The relative content of each mineral in each sample was determined by its proportionate diffraction intensity, i.e. the diffraction intensity of each mineral measured in counts per second (cps) divided by the sum of the diffraction intensity (in cps) of all chosen minerals. To enable further mineralogical characterization, we additionally calculated XRD peak ratios related to the elemental composition of Phyllosilicates. Low values (<0.1) of the 5/10 Å ratio (Esquevin-Index [35], further denoted as EI) indicates iron-rich biotitic composition of micas, while values >0.5 are related to the dominance of micas with Al-rich muscovitic composition [35]. The ratio (7.0 Å + 3.54 Å) / 4.72 Å, further denoted as CR, is used to distinguish chlorites, with values >10 indicating high iron concentrations, while values around 2.4 stand for no iron in octahedral sites in the crystal lattice [36].

Analysed materials comprised 58 reference samples from alluvial fans in the catchment that were used to recognize and characterize source regions of the main inflows. In addition, 28 lake surface samples over the whole lake areas and along depth transects were used to infer the spatial patterns of sediment dispersal in the modern lake system. The temporal pattern of variable sediment supply was inferred from 151 samples, taken from three vertically overlapping sediment cores PG2059, PG2060 and PG2061.

**Supplementary Table 1:** Chosen reflection angles for major mineral occurrence in the study area.

| **Mineral** | Quartz | Plagioclase | Feldspar | Hornblende | Mica | Chlorite | Illite | Dolomite |
|---|---|---|---|---|---|---|---|---|
| **d value [Å]** | 3.343 | 3.192 | 3.245 | 8.43 | 9.97 | 7.06 | 3.57 | 2.888 |
| **2 Theta [°]** | 26.62° | 27.94° | 27.50° | 10.52° | 8.78° | 12.54° | 24.86° | 30.96° |

## 1.2 Grain size analysis

Hydraulic size-density sorting of mineral grains is known to systematically distort quantitative provenance analysis [51]. To assess a potential grain size bias on the semi-quantitative provenance approach presented in this study, we analysed grain size variations of lacustrine sediments. The grain size analysis followed the analytical procedure e.g. described in [41]. Prior to grain size measurements, core samples were treated with acetic acid ($CH_3COOH$) and placed on a sample shaker for 24 h. Additionally, organic components were removed by adding 35 % $H_2O_2$ to sediment samples. The sand fraction was separated by wet sieving using a 63 µm mesh. The separation of the silt to sand fraction was applied by the Atterberg method using settling tubes. Subsequently, sediments of all grain size fractions were dried and weighed. Grain size distributions for sediments <1 mm were analysed using a laser diffraction particle size analyser Coulter LS 200 (Beckmann Coulter GmbH). Final distributions were calculated using the "Fraunhofer" optical model.

## 1.3 Statistical Provenance Analysis

### 1.3.1 Data Pre-processing

Relative mineral diffraction intensities of the complete data set (basin, lake and core samples) were transformed prior to cluster analysis. For subsequent explanations, imagine an *m* x *n* matrix S with observations organized in columns (S = $x_{o1}$, $x_{o2}$… $x_{om}$) and samples organized in rows (S=$x_{s1}$, $x_{s2}$… $x_{sn}$).

First, a centered log-ratio transformation [37] was applied to avoid spurious correlations because of the compositional nature of the data. After replacing each zero element of the data matrix with $10^{-6}$ of the sample variance to avoid ln(0), we calculated the logarithm of each sample divided by its exponential mean. A minor adjustment to the original formula [37] was made to enable a re-transformation:

$$g_s = \left(\prod_{i=1}^{n} x_{si}\right)^{\frac{2}{n}} \quad (1)$$

In a second step we applied a range transformation to reduce differences in observations between the sample sets. The transformation was applied by:

$$\frac{x_{oi}}{x_{oj_{max}} - x_{oj_{min}}} \quad (2)$$

With $x_{oi}$ being the $i^{th}$ observation in column $j$. This kind of range transformation is known to recover the underlying cluster structure more efficiently compared to other transformation algorithms [38].

### 1.3.2 Cluster Analysis

We applied a Fuzzy C-means (FCM) cluster algorithm [39],[40],[41], on the relative bulk mineralogical content of the basin reference samples. FCM is a well-established clustering algorithm which is based on fuzzy logic [42]. In contrast to crisp clustering algorithms where each sample is assigned with exactly one cluster center, FCM assigns a membership degree $\mu_i$ between 0 (no membership) and 1 (full membership) to a sample $s_i$ for all estimated cluster centers with the restriction that all $\mu$ of a sample sum up to 1. The procedure of FCM partitions the data space into a natural number of sub-sets in an iterative process. In each step a prescribed number of clusters centers is imposed on the data space and iteratively rearranged to minimize an objective function $J$. The final partition is reached when either no significant change in $J$ in between two iterations takes place or the algorithm reaches a prescribed number of iterations. We applied the cluster analyzes with a minimal amount of improvement in $J$ between iterations of $10^{-5}$, a maximal number of iteration of 100 and a fuzzyfier $\beta$ of 2.

### 1.3.3 Cluster Validation

To evaluate a suitable number of cluster center we performed a cluster validation by means of the Xie-Beni index (XBi) [43],[44]. XBi is a measure of cluster separation in terms of cluster distance and inner cluster compactness in terms of membership degrees. Generally, a partition of the data space with minimal XBi indicates an optimal cluster number. Because FCM is often corrupted by local minima in the objective function which can lead to undesired partitioning we performed a multiple simulation approach: for each cluster number between 2 and 9 we applied the FCM algorithm 2000 times. In each simulation, the XBi of the resulting partition was calculated. The partition with the lowest XBi was chosen for subsequent analysis.

### 1.3.4 Similarity to Cluster Center

The similarity of lake surface and piston core samples to the previously determined cluster center was analysed by calculating fuzzy membership degrees. The membership degree (μ) of a sample $s_i$ ($s = x_{s1}, x_{s2} \ldots x_{sn}$) to a cluster $C_j$ ($C = x_{c1}, x_{c2} \ldots x_{cn}$) was calculated using the formula:

$$\mu_{sC} = \left( \sum_{k=1}^{n} \left( \|s_i - C_j\| * \|s_i - C_k\| \right)^{\frac{2}{\beta - 1}} \right)^{-1} \quad (3)$$

With $\beta$ being the fuzzyfier applied in the clustering routine. The latter term norms the sum of the membership degrees of a sample to all clusters to 1.

## 1.4 Barrier Identification

To identify the spatial extent of the North Tibetan ISM barrier we analysed the orographic structure of the North Tibetan Plateau. The analysis was carried out on a digital elevation model (DEM) obtained by the Shuttle Radar Topography Mission (SRTM3) made available by the U.S. Geological Survey. The horizontal resolution of the DEM is 3 arc seconds corresponding to ~90m pixel size with a vertical resolution of 1 m. SRTM3 was chosen because of its global coverage and high accuracy in areas without significant vegetation and hence canopy [45]. We analysed the DEM between 86° and 100°E as well as 35° to 38° N.

In a first step we calculated the positive vertical displacement ($\delta z^{\uparrow}$) from each raster cell to its northern nearest neighbour by:

$$\delta z^{\uparrow} = \begin{cases} |z(i,j) - z(i+1,j)|, & |z(i,j) - z(i+1,j)| < 0 \\ 0, & |z(i,j) - z(i+1,j)| \geq 0 \end{cases} \quad (4)$$

With *z(i,j)* being the elevation value *z* of a raster cell at row *i* and column *j* of the DEM raster.

In a second step we calculated the vertical displacement an air mass experiences while migrating from south to north for a distance required passing the Kunlun range completely ($\delta z_k^{\uparrow}$).

In a third step we classified the $\delta z_k^{\uparrow}$ raster by marking all areas within the DEM showing a similar or higher orographic forcing compared to the Kunlun range in the study area in terms of $\delta z_k^{\uparrow}$.

## 1.5 Meteorological Data

To verify the present day blockage effect of ISM precipitation we calculated mean daily precipitation intensities for the ISM season in the month June, July and August (JJA). We selected 15 meteorological stations directly south and north of the North Tibetan precipitation barrier between 89° and 100° E (Supplementary Table 2). Data are available for the period between 1951 and 1980 [46]. Because of the sparse density of meteorological stations we additionally analysed summer precipitation patterns from the High Asian Reanalysis data set (HAR [33]), available for the period 2002 to 2012 in a spatial resolution of 10 km.

## 1.6 Age-Depth Relationship

A previous study [32] established an age-depth relationship for sediments of the piston core presented in this study. The relationship was analyzed using a process- and provenance-based model to estimate mean sedimentation rates, verified by a total of 19 plant remains (*Potamogeton* spec.) used for $^{14}C$ dating as well as paleomagnetic measurements from a total of three coring locations throughout Lake Heihai. Combining all estimated sedimentation rates, the approach yielded a confidence interval for the reservoir effect corrected ages. For this study we use the maximum age scenario (model 3, see Fig. 5 in [32]) for the age-depth relationship of the piston core. This model is closest to the $^{14}C$ ages observed in the piston core used in this study (see supplementary Fig. 1) with a mean deviation of ~2.5 cal. ka compared to a deviation of ~5.4 cal. ka for the minimum age scenario. Additionally, the maximum age-depth scenario is the only model which accounts for a low reservoir effect during the initial stage in lake development (dates 11 and 12 in supplementary Fig. 1 or S17 and S18 in Fig. 5 in [32]) as suggested by decreased inputs of allochtonous carbonate during this stage [32]. Although the applied age-depth relation leads to a good correspondence of our record with the timing of observed environmental changes throughout monsoonal Asia (see Fig. 4), we emphasize the probabilistic nature of sediment ages in our record and restrict our analysis to millennial time scales.

**Supplementary Table 2**: Chosen meteorological stations on the Northern Tibetan Plateau. Mean summer precipitation is given for the period 1951-1980.

| Name | index | Location relative to Barrier | Latitude | Longitude | Elevation (m a.s.l.) | Mean JJA prec. (mm/d$^{-1}$) |
|---|---|---|---|---|---|---|
| Mangya | 1 | Leeward | 38.21 | 90.13 | 3139 | 34.7 |
| Geermuxiaodu | 2 | Leeward | 36.54 | 93.10 | 2843 | 17.5 |
| Geermuxiaozaohuo | 3 | Leeward | 36.40 | 93.31 | 2771 | 18.2 |
| Geermu | 4 | Leeward | 36.25 | 94.54 | 2808 | 23.7 |
| Geermuchaerhan | 5 | Leeward | 36.48 | 95.18 | 2679 | 17.3 |
| Dolannuomuhong | 6 | Leeward | 36.26 | 96.25 | 2790 | 25.6 |
| Dulanxiaangride | 7 | Leeward | 36.04 | 97.48 | 2905 | 94.9 |
| Dulan | 8 | Leeward | 36.18 | 98.06 | 3191 | 102.5 |
| Wulanchaka | 9 | Leeward | 36.47 | 99.05 | 3088 | 128.6 |
| S02 | 10 | Windward | 35.17 | 89.04 | 4910 | 197 |
| Geermuwudaoling | 11 | Windward | 35.13 | 93.05 | 4612 | 183.9 |
| Qumalai | 12 | Windward | 34.,33 | 95.29 | 4231 | 241.3 |
| Madue | 13 | Windward | 34.55 | 98.13 | 4272 | 190.8 |
| Maqingrenxiamu | 14 | Windward | 34.16 | 99.12 | 4211 | 254.2 |
| Guoluo | 15 | Windward | 34.28 | 100.15 | 3719 | 307.9 |

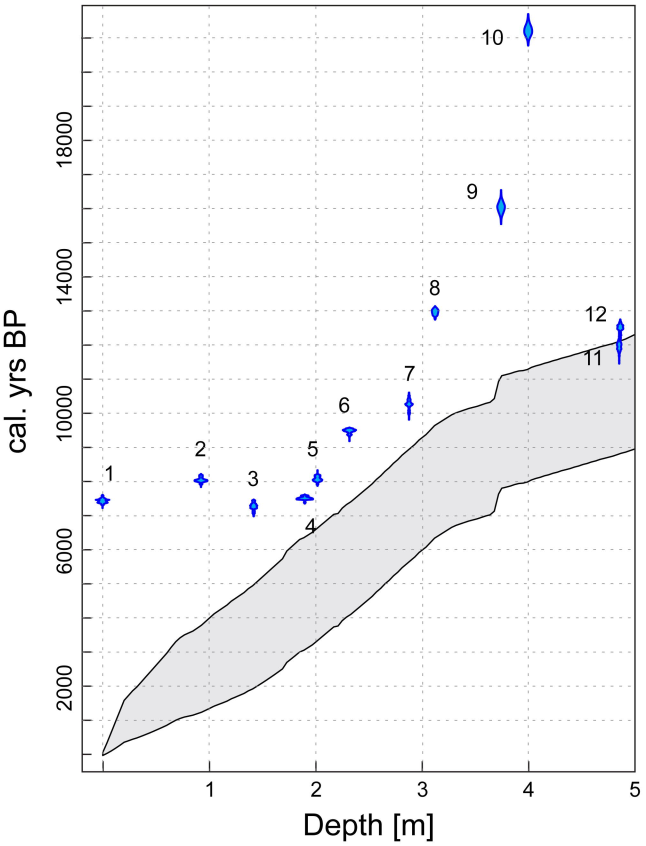


**Supplementary Figure 1**: Age-depth relation of piston core sediments. Blue areas delineate the 95% interval of measured $^{14}C$ ages from plant remains within the core sediments. The confidence interval for reservoir corrected ages inferred by [32] is illustrated as grey area. Black lines around the confidence interval correspond to the maximum and minimum age scenarios.

# 2 Supplementary Results and Discussion

## 2.1 Mineralogical Cluster

Our multiple simulation approach to find an optimal cluster number yielded multiple local minima in XBi for 2 to 10 cluster (supplementary Fig. 2). We chose the global minimum in XBi of 4 clusters with a value of 0.1217 apparent 33.9% of simulations. The corresponding cluster center and their relative mineral contents are shown in supplementary Fig. 3.

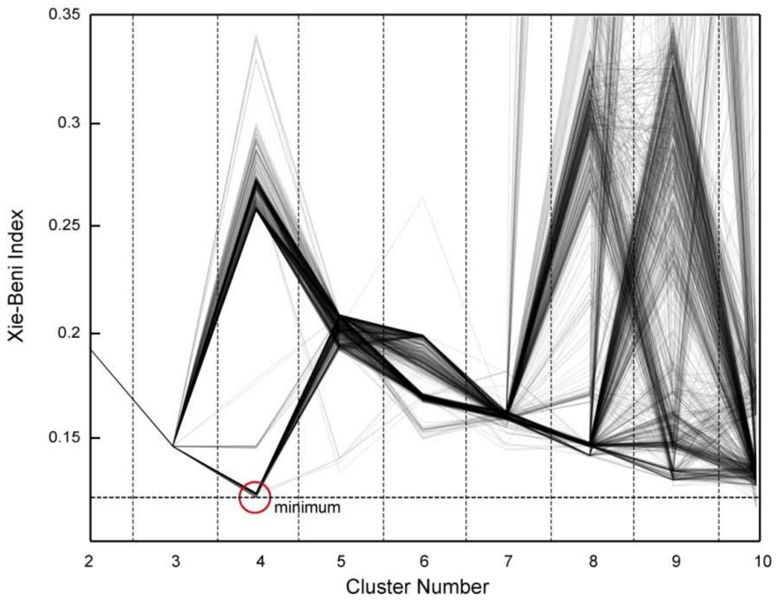


**Supplementary Fig. 2**: Results of cluster validity analysis. Xie Beni indices are shown for cluster numbers 2 to 10. Each grey line indicates one simulation. A red circle shows the global minimum in XBi.

The mineralogical composition of cluster center (supplementary Fig. 3) is in good agreement with the geological setting in the study area (Fig. 2, Supplementary Fig. 4). All cluster center are unique in their mineralogical composition and spatial distribution:

*Cluster 1* (Kunlun range) is mainly characterized by high relative concentrations in Phyllosilicates (chlorite and mica), plagioclase and mica as well as low relative concentrations of alkali-feldspar and hornblende. Its spatial occurrence is restricted to alluvial fans originating from the ice-capped mountains of the Kunlun range. The ice cap overlies Triassic slate and basalt accounting for the dominance of fine grained minerals in cluster 1. EI ratios above 0.5 suggest Al-rich illites formed by strong hydrolysis [47]. Hydrolysis was reported to be responsible for the formation of illite from Alkali-Feldspar under glacial conditions [48], both explaining high illite and low alkali-feldspar concentrations in cluster 1.

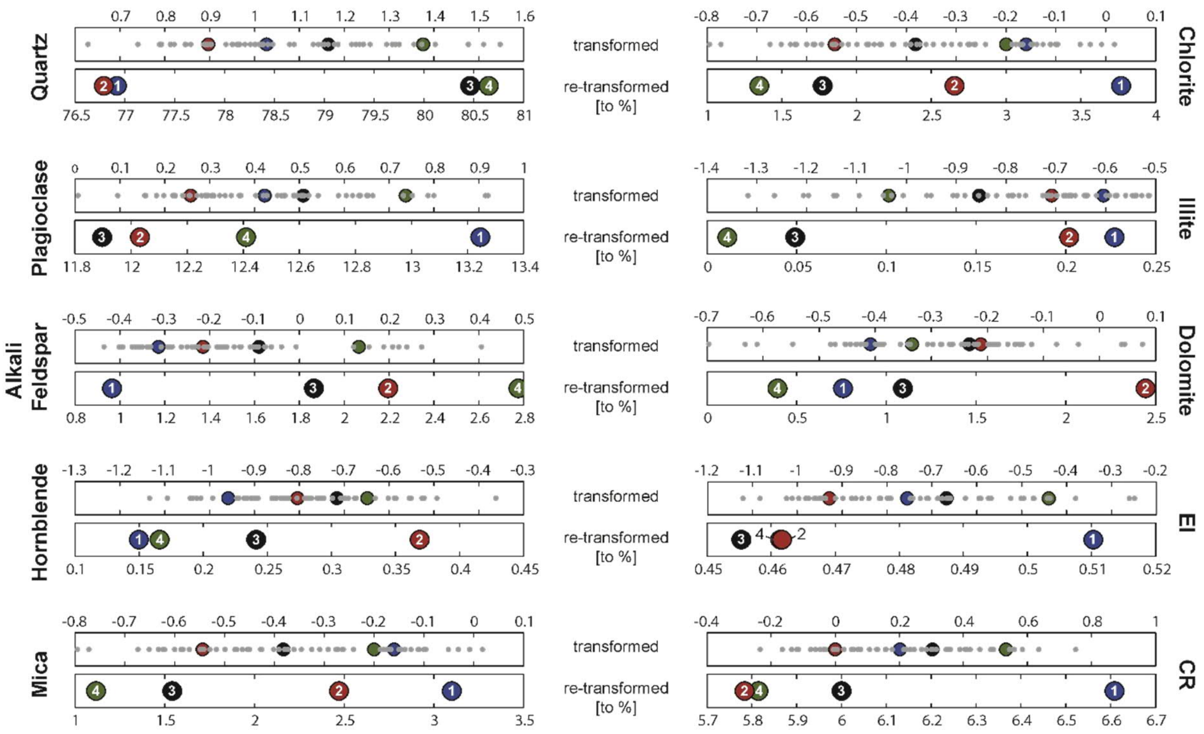


**Supplementary Fig. 3**: Mineralogical cluster. The figure shows transformed and retransformed cluster center for each mineral and two peak area ratios EI and CR. Cluster center are indicated by coloured circles which are additionally labelled with cluster number for the transformed data. Grey dots show the distribution of transformed reference samples in the cluster structure.

*Cluster 2* (Kunlun range) is mainly characterized by high relative concentrations of Hornblende and Dolomite and low relative concentrations of Quartz. The mineralogy suggests carbonate-rich rock sources. The spatial occurrence is restricted to alluvial fans originating from a Permian Limestone outcrop on the foothills north of the Kunlun range. This is the only area in the catchment with outcrops of limestones, allowing for the supply of allogenic carbonates to the lake.

*Cluster 3* (Burhan Buda range) is mainly characterized by low relative plagioclase and high quartz concentrations, reflecting the widespread occurrence of quartzitic sandstones. Notably, cluster 3 tends to separate cluster 2 from cluster 4 with higher mineralogical similarity to cluster 4. Cluster 3 occurs in close distance to the lake shore with a dominance of alluvial fans originating from the Burhan Buda range.

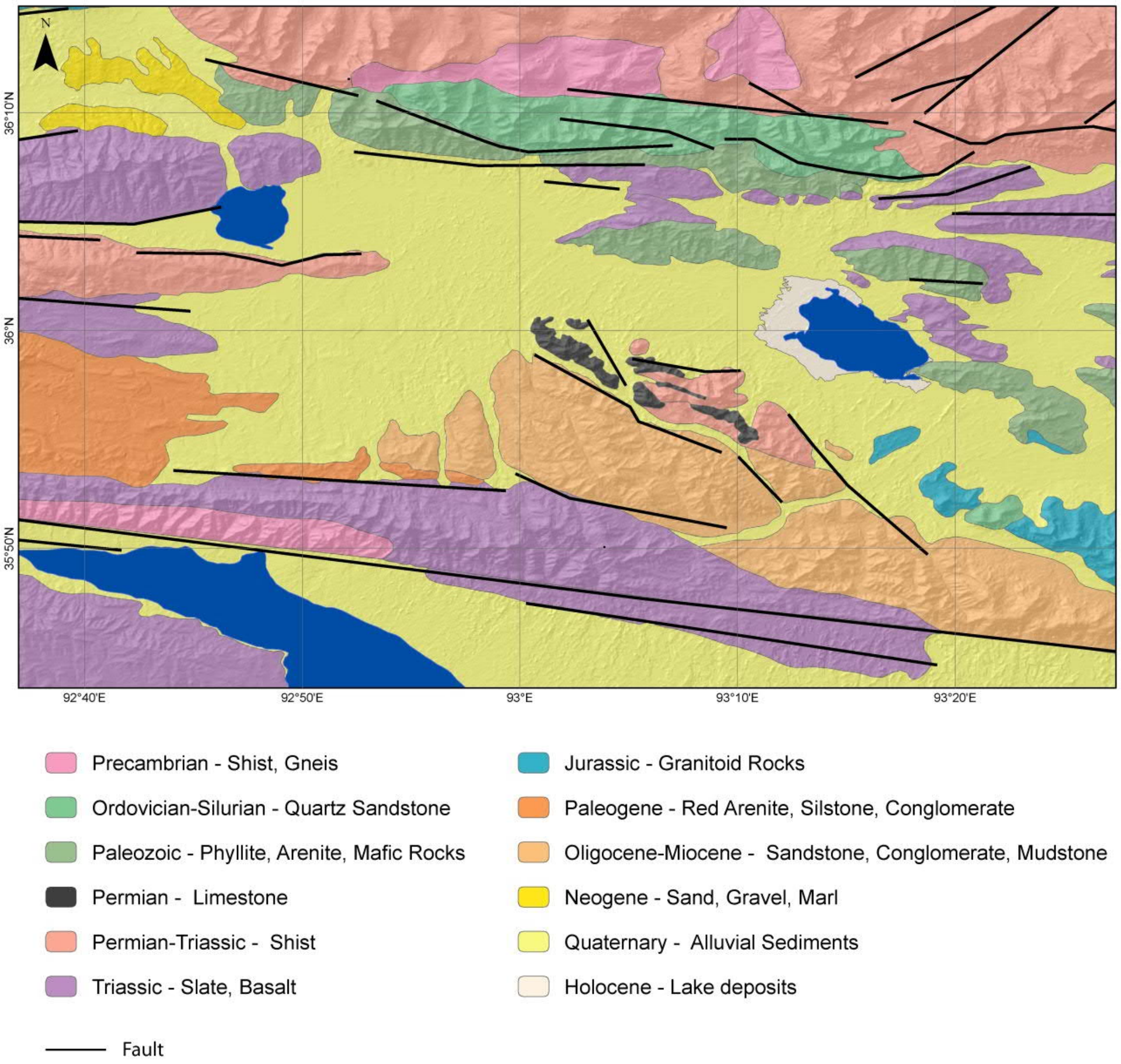


**Supplementary Figure 4**: Geological Map of the study area. The map is adopted and compiled from two existing geological maps [49],[50]. Limestone outcrops in the study area were digitalized from Landsat TM 5 remote sensing data (Band combination 3, 1, 5 RGB). Mapping results were validated by field inspection of outcrops and sampling of rock fragments in the alluvial fans. Map was created using ArcGIS 10.1 (www.esri.com) and Adobe Illustrator CS 4 (www.adobe.com).

*Cluster 4* (Burhan Buda range) is mainly characterized by a low relative content of clay mineral groups and dolomite as well as high quartz and alkali-feldspar contents. Its spatial occurrence is bound to alluvial fans originating from foothills of the Burhan Buda range. The foothills mainly consist of quartz-sandstone and phyllite formations intercalated with Triassic Slate.

The spatial distribution of membership degrees μ to cluster center of basin reference samples (Fig. 2) primarily mirrors geological differences in between the Kunlun and Burhan Buda mountain range. The intra-lake distribution of membership degrees (Fig. 3) shows no

influence of sediments sorting through e.g. current dynamics on the distribution of membership degrees of lake surface samples to mineralogical cluster center. We conclude that mineralogical sediment sources are reliable indicators of sediment provenance over geological time scales in the study area.

## 2.2 The impact of grain size variations on the mineralogical composition of core samples

The siliciclastic component of core samples is mostly comprised of silts with a mean of 63.8 % and variations in between 35.0 and 71.5 %. Sands are the second most abundant component with a mean of 20.0 % and variations in between 6.2 and 60.6 %. The lowest component of siliciclastic grains falls in the clay fraction with 16.2 % on average and variations in between 2.6 and 29.9 %. To analyse a potential impact originating from grain size variations to the abundance of single mineral phases due to e.g. size-density sorting [51], we calculated the correlation coefficient (r) and the coefficient of determination ($r^2$) between grain size classes and the abundance of single mineral phases. The results are presented in supplementary Table 3. Although the overall impact of grain size variations on the abundance of single mineral phases is low with 13 % explained variance on average, there is a tendency of alkali-feldspar and dolomite enrichment in the sand fraction and a mica, chlorite and illite enrichment in the silt to clay fraction.

**Supplementary Table 3**. Correlation coefficients and coefficients of determination (in brackets) between variations grain size classes and mineral phases as estimated by XRD in sediments from core samples.

| | **Quartz** | **Plagioclase** | **Alkali Feldspar** | **Hornblende** | **Mica** | **Chlorite** | **Illite** | **Dolomite** |
|---|---|---|---|---|---|---|---|---|
| **Sand [%]** | 0.06 (0.00) | 0.33 (0.11) | 0.60 (0.36) | 0.11 (0.01) | -0.39 (0.15) | -0.38 (0.14) | -0.48 (0.23) | 0.30 (0.09) |
| **Silt [%]** | 0.04 (0.00) | -0.33 (0.11) | -0.52 (0.27) | 0.02 (0.00) | 0.18 (0.03) | 0.20 (0.04) | 0.24 (0.06) | 0.04 (0.00) |
| **Clay [%]** | -0.15 (0.02) | -0.22 (0.05) | -0.48 (0.23) | -0.22 (0.05) | 0.48 (0.23) | 0.45 (0.20) | 0.58 (0.34) | -0.57 (0.33) |
| **Mean Φ** | -0.22 (0.05) | -0.21 (0.05) | -0.46 (0.21) | -0.17 (0.03) | 0.51 (0.26) | 0.48 (0.23) | 0.60 (0.36) | -0.47 (0.22) |

To assess the impact of selective mineral enrichment in different grain size classes on the provenance signal, we additionally calculated correlation coefficient (r) and the coefficient of

determination ($r^2$) between variations in grain size classes and the similarity of a sediment sample (measured in µ) to each provenance cluster C1 to C4. The results (see supplementary Table 4) suggest a subordinate influence of grain size variations on the provenance signal with 0.06 shared variance on average. Especially the provenance signal of source areas located on the Burhan Buda range (C3 and C4) is independent from mean and class specific grain size variations. The mineralogical similarity to source areas on the Kunlun range, however, seem to be slightly affected by grain size variations, with similarities to cluster C1 favoured by finer grain sizes (silt to clay) and, to a lesser degree, similarities to cluster C2 favoured by coarser grain sizes (sand). This size-provenance relationship may be explained by an increased abundance of fine grained minerals in cluster C1 like illite, chlorite and mica (see supplementary Fig. 3). We thus assume a subordinate influence of grain size variations on the overall provenance signal.

**Supplementary Table 4**. Correlation coefficients and coefficients of determination (in brackets) between variations in grain size classes and the mineralogical similarity (in µ) to major source areas (cluster C1 to C4).

| | **C1** [µ] | **C2** [µ] | **C3** [µ] | **C4** [µ] |
|---|---|---|---|---|
| **Sand [%]** | -0,40 (0.16) | 0,24 (0,06) | -0,01 (0,00) | -0,05 (0,00) |
| **Silt [%]** | 0,16 (0,03) | -0,06 (0,00) | -0,07 (0,00) | 0,00 (0,00) |
| **Clay [%]** | 0,54 (0,29) | -0,36 (0,13) | 0,09 (0,01) | 0,09 (0,01) |
| **Mean Φ** | 0,52 (0,27) | -0,30 (0,09) | 0,01 (0,00) | 0,01 (0,00) |

## 2.3 Barrier Extent

Supplementary Fig. 5 presents $\delta z_k^{\uparrow}$ values within the spatial extent of the study area. The highest values of orographic forcing on southerly air masses ($\delta z_k^{\uparrow}{}_{max}$) of the Kunlun Mountains are in a range of 272 to 1217 m with a mean of 686 m and a standard deviation of 206 m. The Burhan Buda Mountains impose a similar, but slightly lower forcing in a range of 341 to 971 m with a mean of 668 m and a standard deviation of 113 m.

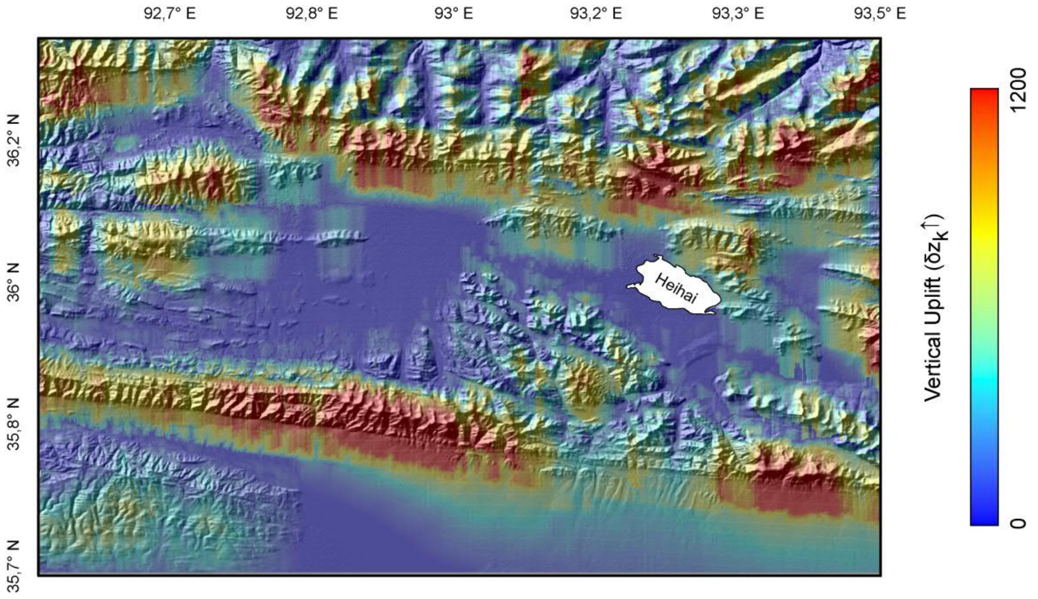


**Supplementary Figure 5**: Vertical Uplift imposed on southerly air masses by the Kunlun and Burhan Buda range as calculated within a 7 km moving window. Map created using Matlab 2008a (www.mathworks.com), ArcGIS 10.1 (www.esri.com) and Adobe Illustrator CS 4 (www.adobe.com).

To enable an identification of the spatial extent of the precipitation barrier we classified $\delta z_k^{\uparrow}$ raster of the Northern Tibetan Plateau according to the orographic forcing within the study area. The results are presented in Supplementary Fig. 6. Since it is uncertain if the lowest orographic forcing imposed by the Kunlun Mountains in the study area could lead to a complete blockage of southerly precipitation when extended laterally, we chose the mean minus the standard deviation as lower classification threshold.

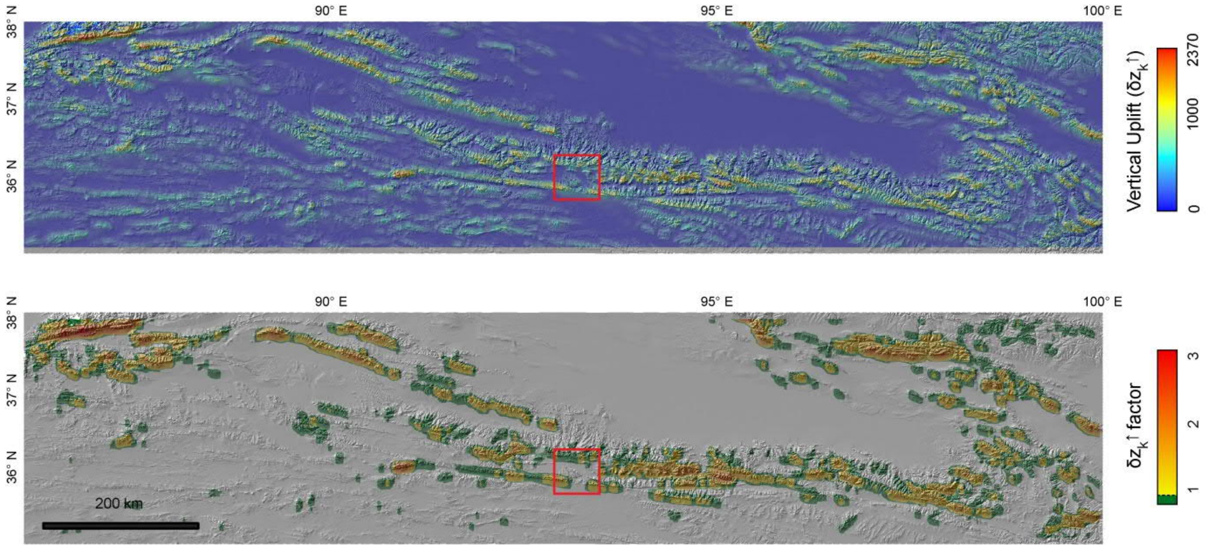


**Supplementary Figure 6**: Spatial extent of the ISM barrier on the North Tibetan Plateau. Red rectangle indicates location of the study area. Up: Vertical Uplift imposed on southerly air masses over the North Tibetan

Plateau. Down: Classification result of $\delta_z k^{\uparrow}$ raster. Green colour indicates lower classification threshold. Yellow to red colours indicate the factor of higher $\delta_z k^{\uparrow}$ values within a moving window (7x7 km) as compared to mean $\delta_z k^{\uparrow}$ of the study area. Maps were created using Matlab 2008a (www.mathworks.com), ArcGIS 10.1 (www.esri.com) and Adobe Illustrator CS 4 (www.adobe.com).

The precipitation barrier extends with similar forcing on southerly air masses for ~1200 km between 86° E and 100°E in between ~36° N and ~38° N normal to ISM trajectories. The barrier encompasses from west to east the eastern Altyn Shan, Qimantag Shan, Kunlun Shan and the western Anyemaquen Shan (Fig. 1).

### 2.4 Modern precipitation patterns on the Northern Tibetan Plateau

Supplementary Fig. 7 presents spatial patterns of summer precipitation intensity on the Northern Tibetan Plateau and adjacent central Asian deserts as calculated from the reanalysed data set [33]. Additionally depicted are the locations of climate stations as presented in supplementary Table 2. Climate stations north of the barrier record ~ 78 % less rainfall on average than climate stations located south of the barrier. The blockage effect is strongest in the western and central parts of the barrier with an approximate blockage of 82 to 91 % of summer precipitation as calculated from the comparison of stations 1 to 6 north and 10 to 12 south of the barrier (see supplementary Fig. 7) and weakest in the eastern parts with a blockage of 48 to 54 % as calculated from the comparison of stations 7 to 9 north and 13 to 15 south of the barrier. A similar trend is apparent in summer rainfall patterns calculated from the reanalysed data set [33]. Although strongly influenced by local topography in detail, rainfall intensities generally drop from ~10 mm in the eastern and central parts as well as ~6 to 8 mm in the western parts south of the barrier to < 1 mm in the Central Asian deserts north of the barrier within a distance of ~150 to 250 km.

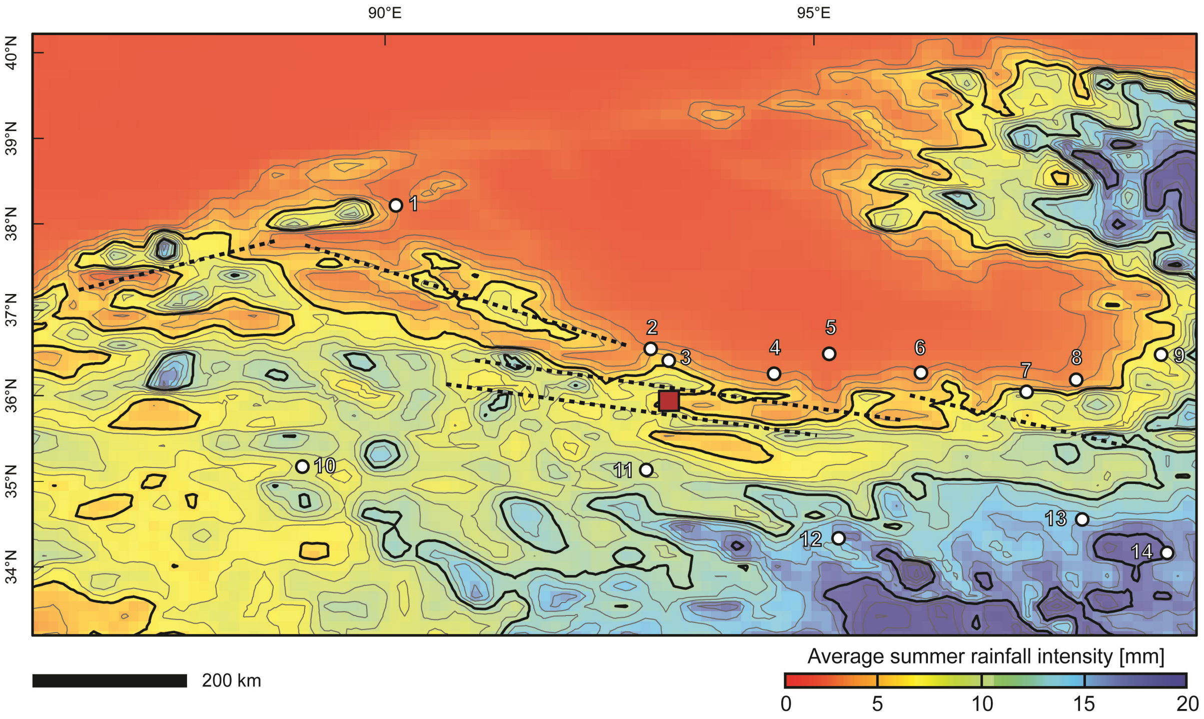


**Supplementary Figure 7**: Modern precipitation patterns on the Northern Tibetan Plateau calculated from HAR reanalysed data [33]. Summer precipitation intensities are presented as coloured raster data and corresponding contour lines. Locations of climate stations are presented as white dots and denoted with indices presented in supplementary Table 2. Dashed black lines denote the extent of the barrier as presented in Fig. 2. Red rectangle denotes the location of Lake Heihai. Map was created using ArcGIS 10.1 (www.esri.com) and Adobe Illustrator CS 4 (www.adobe.com).

## Supplementary References


[34] Biskaborn, B. K., Herzschuh, U., Bolshiyanov, D. Y., Schwamborn, G., Diekmann, B. Thermokarst Processes and Depositional Events in a Tundra Lake, Northeastern Sibiria. Permafrost and Periglac. Process. 24, 160-174 (2013).

[35] Esquevin, J. Influence de la composition chimique des argiles sur la cristallinite. Bull. Centre Rech. Pau – SNPA. 3, 147-154 (1969).

[36] Moore, D. M., Reynolds., R. C. X-ray diffraction and the identification and analysis of clay minerals. 2nd ed. Oxford Univ. Press, New York (1997).

[37] Aitchison, J. The Statistical Analysis of Compositional Data. Chapman & Hall, New York (1986).

[38] Milligan, G. W., Cooper, M. C. A study of variable standardization. J. Classif. 5, 181-204 (1988).

[39] Dunn, J. C. A Fuzzy Relative of the ISODATA Process and Its Use in Detecting Compact Well-Separated Clusters. Journal of Cybernetics 3, 32-57 (1973).

[40] Bzedek, J., Pattern recognition with fuzzy objective function algorithms, Plenum Press, New York (1981).

[41] Opitz, S. et al. Spatio-temporal pattern of detrital clay-mineral supply to a lake system on the northern Tibetan Plateau, and its relationship to late Quaternary paleoenvironmental changes. Catena, 137, 203-218 (2016).

[42] Zadeh, L. A. Fuzzy sets. Inform. Control 8, 338-353 (1965).

[43] Xie, X. L., Beni, G. A validity measure for fuzzy clustering. IEEE Trans. Pattern Anal. Machine Intell. 13, 841-847 (1991).

[44] Wu, K. L., Yang, M. S. A cluster validity index for fuzzy clustering, Patern Recogn. Lett. 26, 1275-1291 (2005).

[45] Braun, A., Fotopoulus, G. Assessment of SRTM, ICESat, and Survey Control Monument Elevations in Canada. Photogramm. Eng. Rem. S. 12, 1333-1342 (2007).

[46] Chinese Meteorological Office, Meteorological Data of China, Meteorology Press, Beijing (1984).

[47] Gingele, F. X., De Deckker, P., Hillenbrand, C.-D. Clay mineral distribution in surface sediments between Indonesia and NW Australia - source and transport by ocean currents. Mar. Geol. 179, 135-146 (2001).

[48] McNamara, M.J. The paragenesis of Swedish glacial clays, GFF, 87, 441-454 (1966).

[49] Kidd, W. S. F. et al. Geological Mapping of the 1985 Chinese--British Tibetan (Xizang--Qinghai) Plateau Geotraverse Route. Phil. Trans. R. Soc. Lond. A 327, 287-305 (1988).

[50] Chengdu Cartographic Publishing House. Geological Map of Qinghai-Xizang (Tibet) Plateau and adjacent Areas. 1:500 000 (2004).

[51] Garzanti, E., Andò, S., & Vezzoli, G. Grain-size dependence of sediment composition and environmental bias in provenance studies. Earth Planet. Sci. Lett. 277(3), 422-432 (2009).
